# Elucidating gene function and function evolution through comparison of co-expression networks of plants

**DOI:** 10.3389/fpls.2014.00394

**Published:** 2014-08-19

**Authors:** Bjoern O. Hansen, Neha Vaid, Magdalena Musialak-Lange, Marcin Janowski, Marek Mutwil

**Affiliations:** Max Planck Institute for Molecular Plant PhysiologyPotsdam, Germany

**Keywords:** comparative transcriptomics, gene function, evolution of function

## Abstract

The analysis of gene expression data has shown that transcriptionally coordinated (co-expressed) genes are often functionally related, enabling scientists to use expression data in gene function prediction. This Focused Review discusses our original paper (Large-scale co-expression approach to dissect secondary cell wall formation across plant species, *Frontiers in Plant Science* 2:23). In this paper we applied cross-species analysis to co-expression networks of genes involved in cellulose biosynthesis. We showed that the co-expression networks from different species are highly similar, indicating that whole biological pathways are conserved across species. This finding has two important implications. First, the analysis can transfer gene function annotation from well-studied plants, such as *Arabidopsis*, to other, uncharacterized plant species. As the analysis finds genes that have similar sequence and similar expression pattern across different organisms, functionally equivalent genes can be identified. Second, since co-expression analyses are often noisy, a comparative analysis should have higher performance, as parts of co-expression networks that are conserved are more likely to be functionally relevant. In this Focused Review, we outline the comparative analysis done in the original paper and comment on the recent advances and approaches that allow comparative analyses of co-function networks. We hypothesize that in comparison to simple co-expression analysis, comparative analysis would yield more accurate gene function predictions. Finally, by combining comparative analysis with genomic information of green plants, we propose a possible composition of cellulose biosynthesis machinery during earlier stages of plant evolution.

## Introduction

The functional annotation of genes is essential for understanding how biological processes are formed, organized, and how they operate. As gene function can mean different things to different people, it is crucial to use controlled vocabulary to define it. To this end, Gene Ontology consortium defined three domains needed to fully describe gene function: Cellular Component (CC—location of gene's activity: e.g., chloroplast lumen, nucleus, small subunit of ribosome), Molecular Function (MF—activity of the gene: e.g., protein binding, protein kinase, carboxylase) and Biological Process (BP—what context is the gene active in: e.g., photosynthesis, protein synthesis, apoptosis) (Ashburner et al., 2000). For example, *Arabidopsis thaliana* cellulose synthase AtCESA1 is active in plasma membrane (Ilic et al., 2007), during cell wall formation (BP), where it has β-(1→4)-glucan synthase activity (MF) (http://www.geneontology.org/). Other popular ontologies include Plant Ontology (anatomy and developmental stages) and Mapman Ontology (visualization of metabolic pathways and other processes) (Thimm et al., 2004; Ilic et al., 2007). While over 40% of the genes in *Arabidopsis thaliana* have at least one of the three domains experimentally revealed, less than 10% of the genes have all three domains verified (reviewed in Rhee and Mutwil, 2014). Therefore, the elucidation of gene function is still one of major hurdles that plant biologists need to overcome.

As the experimental elucidation of function for every gene in Arabidopsis is progressing slowly at current pace, researchers have been turning to *in silico* approaches for assistance in predicting gene function. While a prediction cannot replace experimental proof of gene function, it can be very helpful in suggesting MF, BP, and CC domains of the cryptic gene. Consequently, this can narrow down experiments necessary to verify function. This makes **gene function prediction** one of the most active areas of bioinformatics, with many different flavors of analyses being constantly developed (Radivojac et al., 2013; Rhee and Mutwil, 2014).

KEY CONCEPT 1. Gene function prediction Bioinformatical method than can estimate function of uncharacterized genes by associating them with genes with known function (for a review see, Rhee and Mutwil, 2014).

In this review, we briefly introduce different gene function prediction methods with special focus on comparative co-expression analysis, and its applications in gene function prediction and function evolution.

## Methods for gene function prediction

Prediction methods are based on the guilt by association principle, where genes are linked by some shared characteristics, such as DNA sequence similarity, similar RNA expression levels or protein 3-D structure (Eisen et al., 1998). If an uncharacterized gene is very similar to a characterized gene, the **guilt by association** principle states that they are likely to have same function. Different approaches are applicable to elucidate different domains of gene function (Rhee and Mutwil, 2014). For example, genomic analyses use DNA or protein sequences to annotate genes based on sequence similarity (useful to elucidate MF), or by investigating which families co-evolve through evolution (BP). Protein-protein interaction data can indicate which proteins are likely to be involved in same BP or cellular compartment (BP, CC). It is important to keep in mind that different methods are applicable to elucidate only one domain of gene function. For example, sequence similarity analysis might reveal that a gene has MF of protein kinase, but it does not reveal the targets of the kinase or which BP or CC the kinase is active in. On the other hand, protein-protein interaction data might imply that a gene is a subunit of proteasome (i.e., BP: protein degradation), but it does not reveal the MF of the gene. Consequently, current prediction methods combine various data sources in attempt to simultaneously elucidate multiple domains of gene function (Lee et al., 2010; Kourmpetis et al., 2011).

KEY CONCEPT 2. Guilt by association In gene function prediction, this principle states that the more characteristics (such as sequence, structure, expression, etc.) two genes have in common, the more likely are they to have same function.

**Co-expression analysis** is a popular method in gene function prediction that uses transcriptomic data (in form of microarrays or RNA sequencing data) to group genes according to the similarity of their expression profiles (Usadel et al., 2009). While the analysis is not suitable to reveal MF of a gene, it has been shown that genes involved in same BP and Cellular Compartment tend to have similar expression profiles (Persson et al., 2005; Ryngajllo et al., 2011). Co-expression relationships between genes can be represented as networks, where nodes represent genes and edges (also called vertices or links) represent significant co-expression relationships between genes (Usadel et al., 2009). The network representation provides a convenient, human-readable representation of the many-to-many relationships between genes and is being used by numerous online tools (Usadel et al., 2009). In addition, availability of many mathematical and heuristic methods in network theory can be applied to estimate the properties and quality of the networks (reviewed in Handl et al., 2005). Finally, network-centric methods, such as estimation of enriched (statistically overrepresented) functions of network neighbors or genes within a cluster can be applied (Sharan et al., 2007; Janga et al., 2011).

KEY CONCEPT 3. Co-expression analysis Guilt by association based approach, where genes that have similar mRNA expression profiles across various tissues are assumed to be functionally related.

The caveats of co-expression analysis include large amount of false negatives, as most abundant microarrays for plants are missing ~40% of genes (Mutwil et al., 2011). In addition, the analysis might return erroneous results if a tissue or perturbation relevant for studied BP is missing. For example, if microarrays comprising flower tissues are absent, any query with flower specific genes will return either none or erroneous results. Furthermore, the resolution of observations captured by microarrays is also important. For instance, if the microarray compendia contains microarrays for whole flowers, but not for different organs of flowers (e.g., sepals, petals, carpels, and stamens), performing a query with a petal-specific gene will likely return flower-specific genes instead. Finally, intuitively, the analysis works best for genes under strong transcriptional control, but *a priori* knowledge if this is the case for the gene of interest is often missing. Recent study has shown that predictions involving primary and secondary metabolism pathways perform much better than predictions in hormonal regulation or cell wall biosynthesis (Kleessen et al., 2013). Nonetheless, the analysis has been successfully applied numerous times to elucidate new members of biological processes, including cell walls (Persson et al., 2005; Maeda et al., 2011; Han et al., 2012).

## The benefits of comparative analyses

Shortcomings of co-expression analysis can be partially remedied by extracting analogous co-expression network from multiple species. The principle behind such analysis is that biologically relevant associations are likely to be independently observed in the different species, whereas false associations are less likely to be repeatedly observed. Indeed, sets of genes that are conserved at both sequence and expression levels among multiple species are expected to play a key role in biological responses (Stuart et al., 2003). Therefore, comparative analysis can be thought as biologically meaningful approach to remove false positives (present due to noise in the data) and false negatives (due to missing data in one of the species).

**Comparative co-expression analysis** is beneficial for several reasons. First, biologically irrelevant relationships generated by noise in the data are not likely to reappear multiple times in the co-expression networks in different species. Hence, the number of false positives should be decreased by inclusion of more analogous networks in the analysis. Second, high-quality co-expression networks might help improve poor co-expression networks, decreasing number of false positives. For example, a co-expression network representing detailed atlas of tissues (e.g., sepals, petals, carpels, and stamens) might help resolve a less detailed network (e.g., consisting of whole flowers only). Third, the comparative analysis provides a more powerful method to transfer functional information from a model organism (such as *Arabidopsis*), to other species. Since comparative co-expression analysis combines co-expression (capable to elucidate BP and CC) with sequence similarity analysis (capable of elucidating MF), all three domains of gene function are interrogated simultaneously. Comparative co-expression analysis can therefore suggest a gene that has the same sequence and the same co-expression profile between species, producing a much stronger prediction than the individual analyses. It is important to keep in mind that the species that are being compared should contain the studied BP. Obviously, comparison of co-expression networks representing photosynthesis is feasible between Arabidopsis and rice but not between *Arabidopsis* and *E. coli*.

KEY CONCEPT 4. Comparative co-expression analysis A method to extract relevant prediction by emphasizing co-expression relationships found independently in multiple species.

There are now numerous tools that allow comparative co-expression analyses (reviewed in Movahedi et al., 2012). Examples include Co-expressed biological Processes (CoP) (Ogata et al., 2010), expression context conservation (ECC) (Movahedi et al., 2011), Gene Co-Expression Analysis Toolbox (GeneCAT) (Mutwil et al., 2008), Plant Network (PlaNet) (Mutwil et al., 2011), STARNET2 (Jupiter et al., 2009), and Expressolog Tree Viewer (Patel et al., 2012). The tools, with exception of PlaNet, compare the co-expression networks between species in a pairwise manner. PlaNet has an additional feature of being able to combine and display information about conserved networks in multiple species. While the original paper that this review is addressing was based on output of PlaNet, in here we are performing a manual analysis. The script to make the analyses is available from http://aranet.mpimp-golm.mpg.de/download/frontiers2014.zip.

## Case study: cellulose biosynthesis in plants

Biosynthesis of plant cell walls has received much attention from bioinformatics (Brown et al., 2005; Persson et al., 2005; Mutwil et al., 2008; Ruprecht et al., 2011). Plant cell walls function as a cellular exoskeleton that defines cell shape and functions as a barrier against environmental threats (Somerville, 2006; Liepman et al., 2010). The cell wall is composed mainly of carbohydrate-based polysaccharides, such as cellulose, hemicelluloses, and pectins, along with polyphenolic lignins, and various glycosylated proteins. Cell walls have been classified into primary cell walls (PCW) and secondary cell walls (SCW), largely depending on the wall function and composition (Carpita et al., 1997). While the PCW in higher plants consists of cellulose, hemicelluloses, and pectins, SCW mainly contains cellulose, xylans, and lignin.

The polysaccharides and glycoproteins, with the exception of cellulose, are synthesized as oligomeric structures in the Golgi, and are subsequently transported to the cell surface where they are incorporated into the cell wall (Geisler et al., 2008). These oligomers are assembled by various glycosyltransferases, potentially working as larger protein complexes during synthesis (Lerouxel et al., 2006; Scheller and Ulvskov, 2010). Cellulose is synthesized at the plasma membrane by multimeric cellulose synthase (CESA) complexes (Somerville, 2006). The CESA complexes consist of three different CESA proteins. Consequently, the CESA-complex that is active during PCW formation consists of the CESA-1, -3, and -6-related proteins (Desprez et al., 2007), while the SCW complex consists of three CESA-4, -7, and -8 (Turner and Somerville, 1997). PCW CESAs, and consequently new cellulose microfibrils co-align with microtubules (Paredez et al., 2006), due to POM2 mediating interaction between CESAs and microtubules (Gu et al., 2010; Bringmann et al., 2012). While many proteins important for PCW and SCW formation are already known, new players are being constantly discovered (McFarlane et al., 2014).

In addition, it has been shown that both PCW and SCW CESAs, can be used as baits to find other genes associated with cell wall production via co-expression analysis (Brown et al., 2005; Persson et al., 2005). These studies revealed genes involved in xylan and lignin synthesis were transcriptionally coordinated with the SCW CESAs. Similar approaches have been applied to synthesis of the PCW hemicellulose xyloglucan (Cocuron et al., 2007). Cocuron et al. (2007) showed that the *Arabidopsis* AtCSLC4 gene, which is presumably involved and synthesizing glucan backbone for the xyloglucan, was co-expressed with other genes associated with xyloglucan synthesis (Liepman and Cavalier, 2012). Furthermore, an analysis of transcriptional coordination of cell wall-related gene families in Arabidopsis revealed that members of some of the gene families tend to be co-expressed, e.g., different chitinase family members tend to be transcriptionally associated with different CESA members (Mutwil et al., 2009). In total, identification of at least eight new genes associated with the cell wall growth is credited to the co-expression analysis (Brown et al., 2005; Persson et al., 2005; Ruprecht et al., 2011).

To illustrate how to manually perform comparative co-expression analysis, we have downloaded co-expression networks of Arabidopsis and rice from PlaNet (http://aranet.mpimp-golm.mpg.de/download/). The networks comprise 21,159 and 39,109 genes for Arabidopsis and rice, respectively. To isolate co-expression networks involved in PCW and SCW biosynthesis, AtCESA-1,-3-6, AtCESA-4,-7,-8, and corresponding PCW and SCW CESAs from rice were used as queries for the networks (Ruprecht et al., 2011). Next, to extract genes associated with cell wall biosynthesis, all nodes (genes) within two steps of the CESAs were collected. In total, 362 and 261 PCW genes and 111 and 122 SCW genes were found from *Arabidopsis* and rice, respectively. The PCW networks are larger, due to more ubiquitous expression profiles of the genes. This is in contrast to SCW-related genes, which are mostly expressed in stems and roots (Mutwil et al., 2008). These networks were used for the following analysis.

## Conservation implies relevance

**Figure 1:**
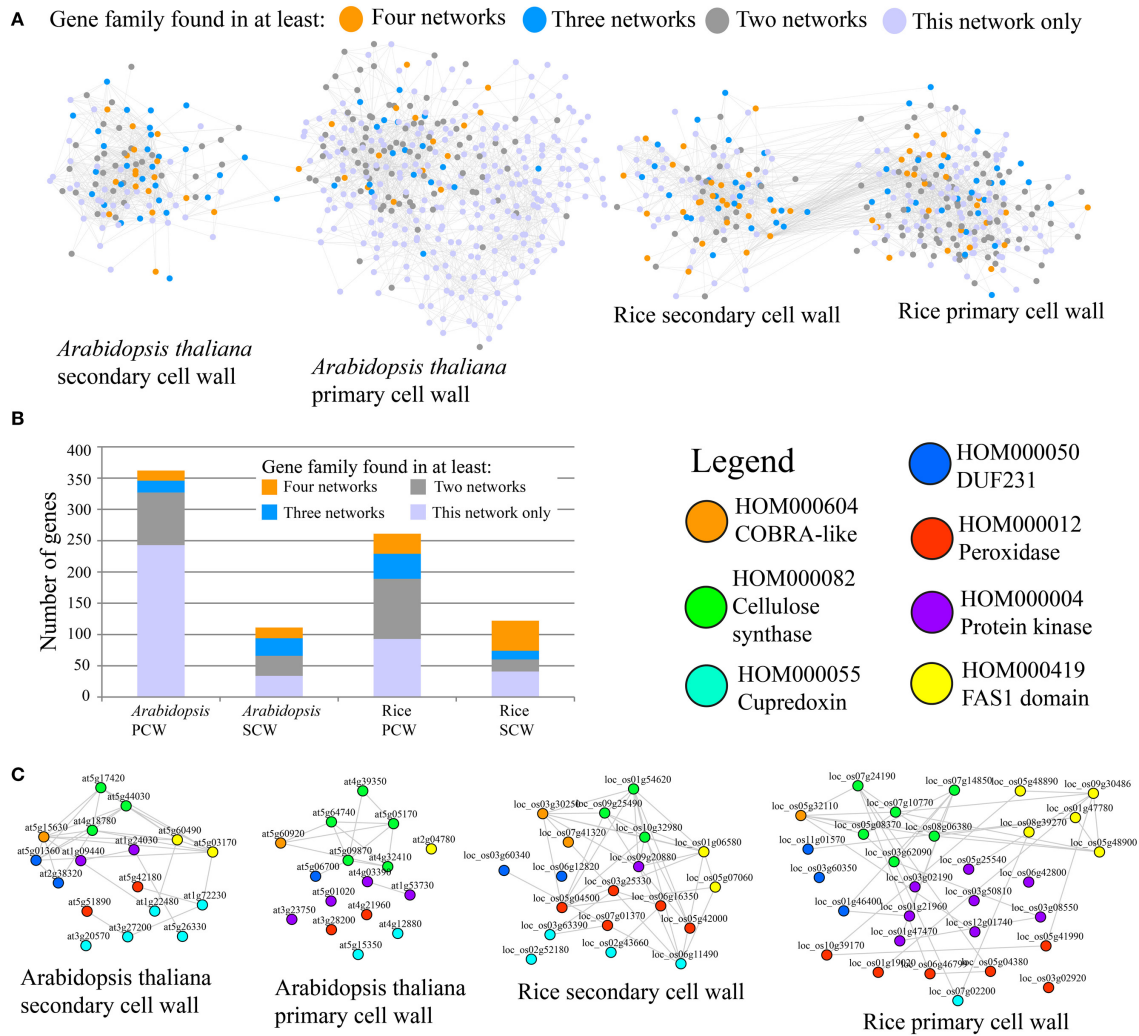
**Comparative co-expression networks of cellulose biosynthesis**. **(A)** Co-expression networks of Arabidopsis and rice primary secondary cell walls. Nodes and edges represent genes and co-expression relationships between genes, respectively. Node colors indicate degree of conservation of families present in the four networks (legend). **(B)** Distribution of conservation classes in the four networks. The y-axis represents total number of genes, while the x-axis represents the four analyzed cell wall networks. Color of the bars depict degree of conservation. **(C)** Filtered PCW and SCW networks where genes that belong to families present in all four networks are shown. Nodes are color-coded according to the family they belong to.

To compare *Arabidopsis* and rice PCW and SCW co-expression networks in terms of similarity, occurrence of gene families defined by PLAZA (http://bioinformatics.psb.ugent.be/plaza/), was measured (Figure 1). It is important to note that in original paper we have used PFAM domains to classify genes into gene families, but we have recently found that PLAZA classifiers perform better (Mutwil et al., submitted). The comparison was carried out by counting the number of networks a given family was present in. For example, since each of the four networks contains CESA family, the family should be counted four times. The result of the analysis can be seen on Figure 1 and Table 1.

**Table 1:** **Annotation of the families enriched in the four networks**.

**Number of times present**	**PLAZA family**	**Description**	**Function**
4	HOM000004	Protein kinase	Brassinosteroid-mediated root growth (Kim et al., 2013)
4	HOM000012	Peroxidase	Associated with lignification (Sato et al., 2006)
4	HOM000050	DUF231	Associated with pectin esterification (Bischoff et al., 2010)
4	HOM000055	Cupredoxin	GPI-anchored electron carrier, relation to cell wall unknown
4	HOM000082	Cellulose synthase	Cellulose biosynthesis
4	HOM000419	FAS1 Domain	GPI-anchored glycoprotein, mutants display reduced strength and altered cell wall architecture in mutants (MacMillan et al., 2010)
4	HOM000604	COBRA-like	GPI-anchored protein of unknown function, mutants display large decrease in cellulose content (Brown et al., 2005)
3	HOM000007	MYB transcription factor	Induces secondary cell wall formation (Zhong et al., 2007, 2008)
3	HOM000013	C3HC4 RING-type	Zinc ion binding, relation to cell wall unknown
3	HOM000017	Serine/threonine/tyrosine-protein kinase	Cell wall integrity-sensing kinases (Hematy et al., 2007; Duan et al., 2010)
3	HOM000037	Peptidase aspartic	Associated with elongating cells (Irshad et al., 2008)
3	HOM000058	Peptidase C1A, papain	Cysteine proteinases superfamily protein, aids in the regulation of autolysis of xylem tracheary elements (Avci et al., 2008)
3	HOM000062	HXXXD-type acyl-transferase	Biosynthesis of lignin (Hoffmann et al., 2004)
3	HOM000086	Lipase, GDSL	Relation to cell wall unknown
3	HOM000088	Fucosyltransferase	Putative fucosyltransferase (Hansen et al., 2012)
3	HOM000188	Glycosyl transferase, family 8	Involved in synthesis of hemicelluloses (Orfila et al., 2005)
3	HOM000228	IQ calmodulin-binding region	Relation to cell wall unknown
3	HOM000272	Chitinase-like1/Pom-Pom1	Mediates binding between cellulose and hemicelluloses (Sanchez-Rodriguez et al., 2012)
3	HOM000285	DUF568	Relation to cell wall unknown
3	HOM000490	DUF250	UDP-galactose transporters, relation to cell wall unknown
3	HOM000515	FAS1 Domain	GPI-anchored glycoprotein, reduced cell elongation in mutants(Lee et al., 2005)
3	HOM000572	Late embryogenesis abundant, LEA2	Hydroxyproline-rich glycoprotein, function unknown
3	HOM000578	DUF869	Relation to cell wall unknown
3	HOM000646	DUF597	4-O-methylation of glucuronic acid on xylan (Lee et al., 2012)
3	HOM000650	Ubiquitin	Apoptosis regulator, relation to cell wall unknown
3	HOM000818	Unknown	Directional control of expanding cell, microtubule interacting (Sedbrook et al., 2004)
3	HOM000854	DUF1218	Relation to cell wall unknown
3	HOM000945	Late embryogenesis abundant, LEA2	Hydroxyproline-rich glycoprotein, function unknown
3	HOM001006	Exostosin-like	Gucuronoxylan synthesis (Brown et al., 2009)
3	HOM004952	Unknown	Relation to cell wall unknown
2	HOM001703	KORRIGAN	β-(1→4)-glucanase, mutants produce aberrant xylem vessels (Szyjanowicz et al., 2004)
2	HOM000137	POM2/CSI	Mediates interaction between CESA complex and microtubules (Gu et al., 2010; Bringmann et al., 2012)

A representation of the four co-expression networks is show in Figure 1A. The nodes (genes) are labeled according to the frequency of the gene family it belongs to. A large number of gene families were present in two, three, or four of the networks (Figure 1A). Apart from *Arabidopsis* PCW network, more than half of genes belong to conserved families, with SCW networks being especially conserved (Figure 1B). Though many of the highly conserved families have been implicated in cell wall biosynthesis, several of the families at the moment have no known function, and are good candidates for functional characterization (Table 1). A highly conserved core of genes belonging to families present in the four networks is shown in Figure 1C. Interestingly, many of the genes in conserved networks are potentially redundant, due to high similarity of gene sequence and expression profiles. For example, each network contains more than one gene belonging to peroxidase family. Uncovering a knock-out phenotype of the peroxidases might necessitate generation of multiple peroxidase knock-outs (Figure 1C).

It is important to note that while many of the highly conserved families are important for cell wall formation, known complex members of the CESA complex are not among the most highly conserved (Table 1). Known complex members include POM2/CSI and KORRIGAN (McFarlane et al., 2014). POM2 (HOM001703) mediates interaction of PCW CESA complex with the microtubules (Gu et al., 2010; Bringmann et al., 2012) and is found to be associated with PCW networks only (Table 1). KORRIGAN (HOM000137), a putative β-(1→4)-glucanase, is similarly found to be associated with PCW networks exclusively (Table 1). Whether or not SCW CESA complex too is interacting with POM2 and KORRIGAN (or their equivalents) is currently unknown and not revealed by the analysis. Furthermore, many of the top conserved families are not directly involved in cellulose biosynthesis, but rather represent various processes that together are important for PCW and SCW formation (e.g., production of hemicelluloses and lignins).

**Figure 2:**
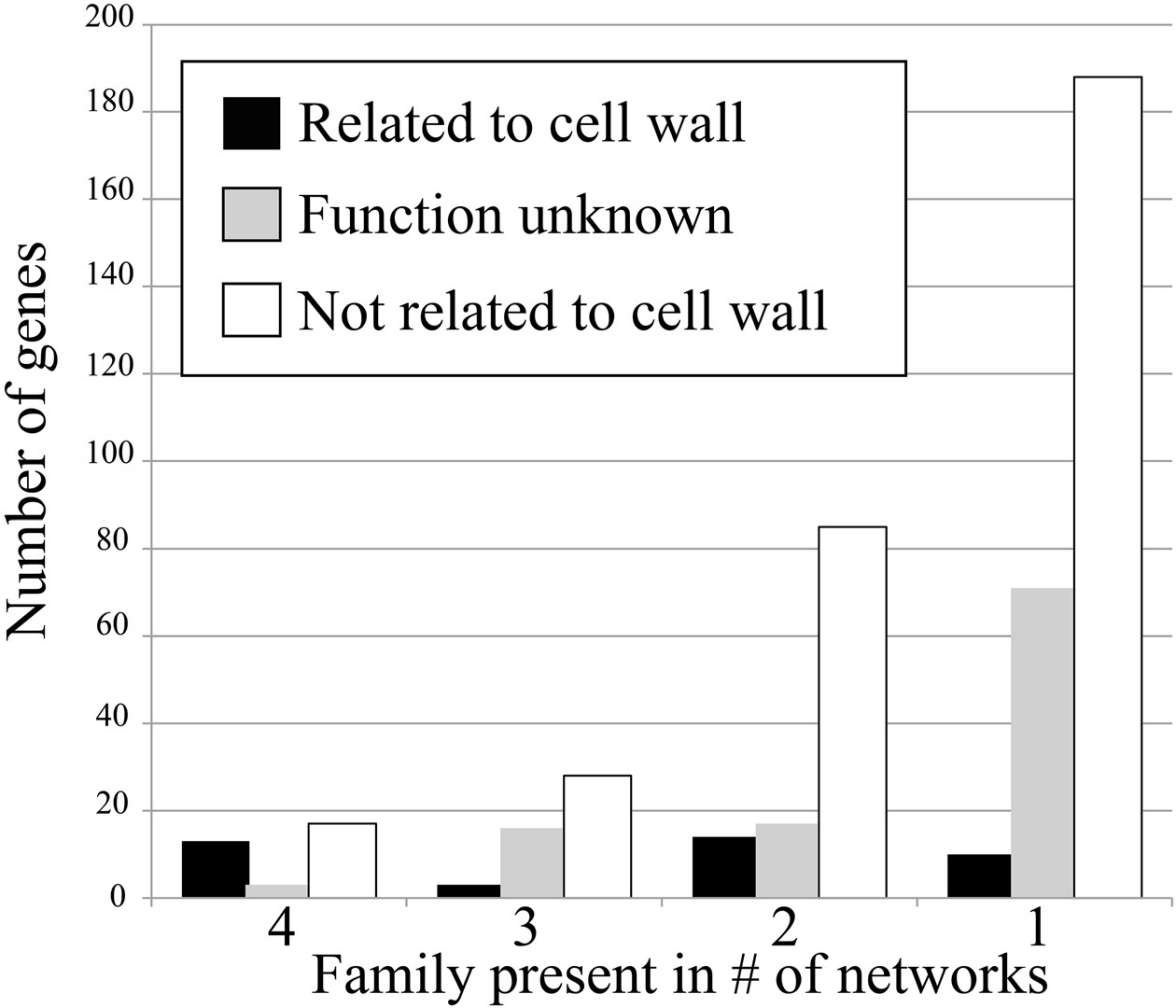
**Number of cell wall-related genes in the four conservation classes**. Genes that are cell wall related (Mapman ontology term 10) are shown in black, genes with unknown function (term 35) are shown in gray, and genes not related to cell wall (any term but 10 and 35) are shown in white.

To test how conservation of gene families in networks corresponds to their relevance, we have counted the amount of cell wall relevant genes present in the conserved families. This was done by counting number of genes annotated with Mapman ontology term 10 (“cell wall”) and 35 (“unknown”). The results show that when the degree of family conservation decreases from four to one, the number of genes that are not relevant for cell wall biosynthesis dramatically increases (Figure 2, denoted by white bar). Therefore, we conclude that that highly conserved families are more functionally relevant, which is in line with studies carried out in humans, flies, worms, and yeast (Stuart et al., 2003).

## Ancestral reconstruction of cellulose synthase network

As more plant genomes are becoming available, comparative genomics are increasingly being used by researches to address some of the major questions in developmental plant biology. Whole plant kingdom has descended from a eukaryotic ancestor that acquired a photosynthetic cyanobacterium as an endosymbiot (reviewed in Bowman et al., 2007 and Banks, 2009). Plants consist of three distinct groups: rhodophytes (red algae), the glaucophytes (little-known freshwater algae), and the green plants (green algae and land plants). The rhodophytes are marine algae that comprise reef-building coralline algae, and provide a source of agar and billion-dollar nori industry in Japan. The highly diverse green plants make up two major clades: the chlorophytes (freshwater and marine algae) and the streptophytes (land plants and paraphyletic charophycean freshwater algae). The land plants pioneered and dominated the land and provided a platform for subsequent colonization of the land surface.

**Figure 3:**
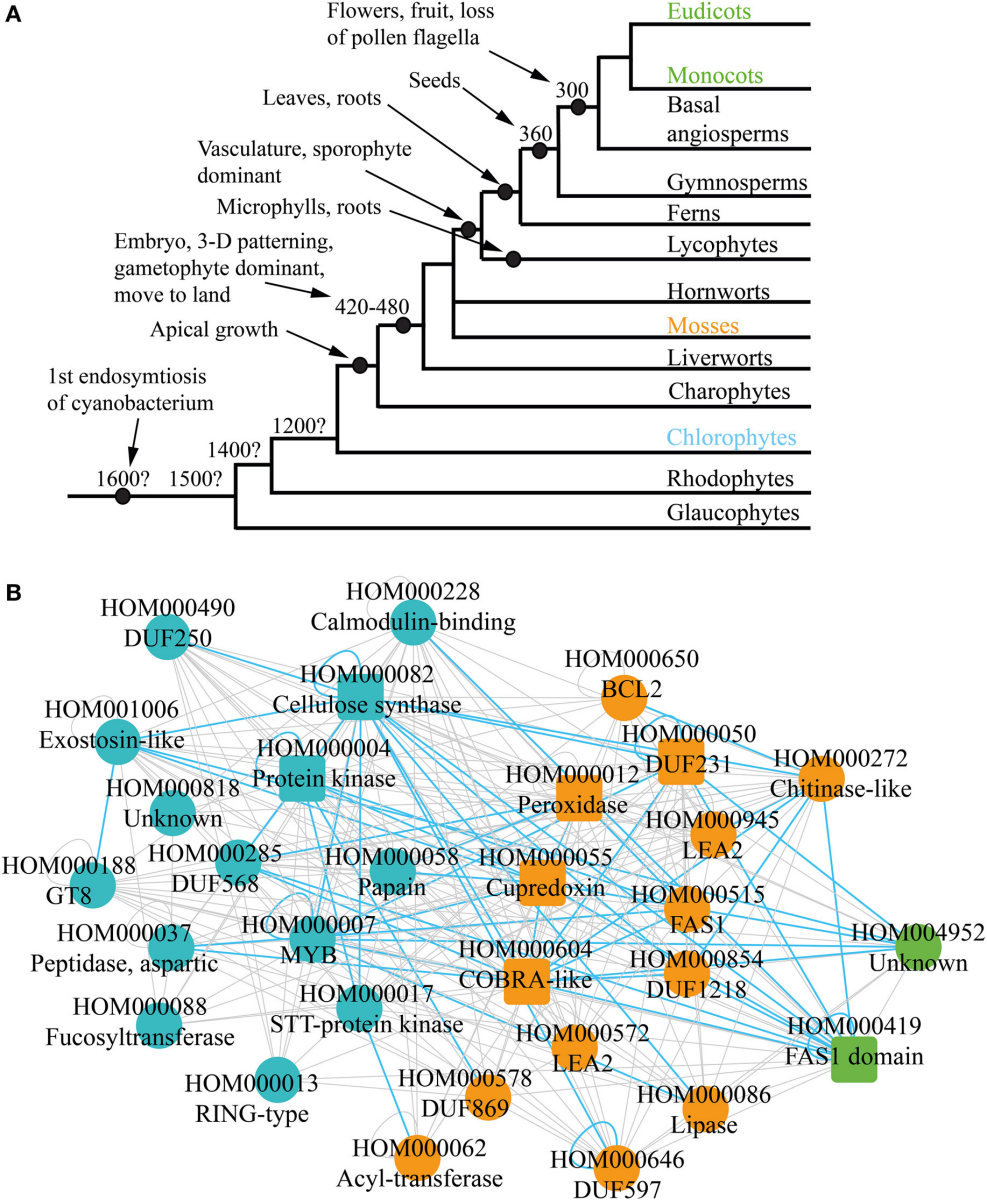
**Phylogenetic analysis of the co-expression networks. (A)** Depicted are relationships among the lineages of plants. Estimated dates for some nodes are shown in millions of years before the present date. Major events are demarcated by black nodes and arrows. **(B)** Gene family consensus network depicting first appearance of the conserved families. Blue, orange, and green represent appearance in charophytes, mosses, and monocots and eudicots, respectively. Square and round nodes represent families present in four and three of the networks, respectively.

Plants underwent multiple revolutionary changes since the endosymbiosis of the cyanobacterium some 1.6 billion years ago (Bowman et al., 2007) (Figure 3A). These include, among others, multicellularity (King, 2004; Ruiz-Trillo et al., 2007), move to land, apical growth (Ueda and Laux, 2012), development of vasculature (Banks, 2009) and flowers (Adams, 2013). Current comparative genomic analyses can indicate which morphological features of plants are associated with emergence or loss of gene families. However, the analyses are based on static genomic data and are investigating functional association of individual genes. It would be therefore beneficial to combine comparative genomic data with comparative transcriptomic data, to elucidate evolution of biological pathways.

While cellulose biosynthesis co-expression networks have been studied extensively in angiosperms (Brown et al., 2005; Persson et al., 2005; Mutwil et al., 2008; Ruprecht et al., 2011), not much is known about the networks in older lineages of green plants, as transcriptomic data for non-angiosperms is scarce. However, it is possible to suggest an ancestral network by combining the four cell wall networks, and retaining only conserved relationships. Apart from indicating conserved gene families, such “consensus” network can show conservation of associations between families. For example, CESA genes are present in all four networks and are always co-expressed with each other (green nodes on Figure 1C). This re-occuring transcriptional association is represented with conserved self-loop (Figure 3B, demarcated by blue loop). Conserved transcriptional associations are also observed between CESA, COBRA, MYB, and other families (Figure 3B, depicted by blue edges). Since the consensus network is obtained by comparing transcriptomic and genomic data from monocots and dicots, we hypothesize that it approximates cellulose synthase network as it was present in one of the ancestors of angiosperms.

Comparative genomic analyses observe presence and absence of gene families in the major lineages of plants (Van Bel et al., 2012). For example, if a family is not present in chlorophytes, but can be found in mosses and angiosperms, one can assume that the family arose somewhere between chlorophytes and mosses (Figure 3A). This information can be readily mapped onto the ancestral network, to elucidate which parts of the network lack the potential to be found in chlorophytes, mosses, and angiosperms (Figure 3B). Around half of the families are found in chlorophytes (blue nodes), and contain many relevant families, such as CESAS, GT8, Exostosin-like, and others (Table 1, Figure 3B). Another half of the network can only be found from mosses on (orange nodes) and also contain many relevant families, such as DUF231, COBRA-like, and FAS1. Surprisingly, very few changes regarding cellulosic wall biosynthesis seem to have happened between mosses and angiosperms, as only two new families have appeared in this period (green nodes). While chlorophytes do produce a cellulose-like polymer mannan by a family similar to cellulose synthases, their cell walls consists mainly of hydroxylproline-rich proteins (Voigt and Frank, 2003; Yin et al., 2009). Since moss cell walls resemble those of higher plants (Roberts et al., 2012), one can speculate that the moss-specific families are associated with biosynthesis of cellulose-rich cell walls found in land plants (Figure 3B).

## Conclusions

Comparative transcriptomic analyses have great potential to elucidate gene function, mediate functional annotation, and study evolution of biological pathways. With steadily increasing amount of transcriptomic and genomic data for non-angiosperms, the conclusions taken from such analyses will improve. Furthermore, the analyses presented here are not limited to transcriptomic data, but can easily be fitted to other co-function gene networks. We envision that future analyses will employ co-function networks based on transcriptomic and protein-protein interaction data, spanning from glaucophytes to angiosperms.

### Conflict of interest statement

The authors declare that the research was conducted in the absence of any commercial or financial relationships that could be construed as a potential conflict of interest.
